# The effect of MEditerranean DIet and MINdfulness eating on Depression severity in people with major depressive disorder and obesity (MEDIMIND): a study protocol of a randomised controlled clinical trial with multifactorial design

**DOI:** 10.1017/S0007114525105849

**Published:** 2026-02-28

**Authors:** Alina Moosburner, Mirela-Ioana Bilc, Dennis Anheyer, Alina Schleinzer, Samaneh Rahmdel, Frank Vitinius, Holger Cramer

**Affiliations:** 1 Institute for General Practice and Interprofessional Care, University Hospital Tübingen, Tübingen, Germany; 2 Robert Bosch Center for Integrative Medicine and Health, Bosch Health Campus, Stuttgart, Germany; 3 Department of Psychology and Psychotherapy, University Witten/Herdecke, Witten, Germany; 4 Interfaculty Institute of Microbiology and Infection Medicine, University of Tübingen, Tübingen, Germany; 5 Department of Psychosomatic Medicine, Robert Bosch Hospital Stuttgart, Stuttgart, Germany; 6 Department of Psychosomatics and Psychotherapy, Faculty of Medicine, University Hospital and University of Cologne, Cologne, Germany

**Keywords:** Mediterranean diet, Mindful eating, Depression, Obesity, Randomised controlled trial, Mental health, Complementary medicine

## Abstract

Obesity and depression are highly prevalent diseases that are strongly correlated. At the same time, there is a growing gap in care, and treatment options should be improved and extended. Positive effects of a Mediterranean diet on mental health have already been shown in various studies. In addition to the physiological effects of nutrients, the way food is eaten, such as mindful eating, seems to play a role. The present study investigates the effect of a Mediterranean diet and mindful eating on depression severity in people with clinically diagnosed major depressive disorder and obesity. Participants will be randomised to one of the four intervention groups (Mediterranean diet, mindful eating, their combination and a befriending control group). The factorial design allows investigating individual effects as well as potential synergistic effects of the interventions. The study consists of a 12-week intervention period, where five individual appointments will take place, followed by a 12-week follow-up. The primary outcome is depression severity. Secondary outcomes are remission of depression, assessor-rated depression severity, quality of life, self-efficacy, BMI, waist:hip ratio and body composition; adherence to the Mediterranean diet and mindful eating will also be assessed. Alongside mediator and moderator analysis, a microbiome analysis, a qualitative evaluation and an economic analysis will be conducted. The study investigates an important health issue in a vulnerable target group. It allows to draw valuable conclusions regarding the effectiveness of different interventions and therefore contributes to improving available care options for people suffering from depression and obesity.

Mental health and dealing with mental illness are becoming increasingly important. The point prevalence of mental disorders increased by 48·1 % between 1990 and 2019 and has reached 12·3 % worldwide^(1)^. In Germany, the lifetime prevalence of depression is 11·6 %^(2)^, with major depression as one of the most common forms. Another present emerging burden of health is obesity. Since 1990, the prevalence in Germany has more than doubled; in 2019, 19·0 % of the German population suffered from obesity^(3)^. The COVID-19 pandemic had a major negative impact on both diseases. Calculations result in 53·2 million additional cases of major depression worldwide^(4)^ and a 3 % increase in the prevalence of obesity^(5)^.

Obesity and depression are significantly correlated with each other^(6)^. Both diseases are predictors of the development of the other. The correlation is stronger with higher grades of BMI and higher severity of depressive symptoms^(7,8)^, indicating a dose–response relationship. Besides social factors^(9)^, shared biological mechanisms including genetic, endocrinology and immunological-inflammatory factors and the microbiome can explain the co-occurrence^(10,11)^.

The availability of treatment options for both diseases is inadequate. To receive adequate therapy, personal and financial efforts and long waiting times are often required^(12,13)^. Additionally, 20–40 % of depression patients are considered non-responders for standard treatment^(14)^. Weight reduction programmes often fail to reach clinical relevance, especially in the long term^(15)^. Prevalence increases with decreasing income and education status^(2,3,9)^, and motivation problems and exhaustion, which often go hand in hand with depression^(16)^, further exacerbate the vicious circle. The urge for low-threshold, easily accessible therapy approaches becomes clear.

An uprising field of research is the influence of diet on mental health. The most popular dietary pattern in this context is the Mediterranean diet. Its effect on depression has already been investigated in a number of randomised controlled trials. A significant improvement in depressive symptoms through Mediterranean diet interventions was shown in the SMILES (Supporting the Modification of lifestyle in Lowered Emotional States), HELFIMED (Healthy Eating for Life with a Mediterranean-style diet) and AMMEND (A Mediterranean Diet in MEN with Depression) trials^(17–19)^. A recent meta-analysis shows a moderate, significant effect in the reduction of depression severity^(20)^. Two further meta-analyses show an inverse relationship between adherence to the Mediterranean diet or a healthy dietary pattern and the onset of depression^(21,22)^.

Regarding obesity, many health benefits of the Mediterranean diet have been described, including weight loss, the reduction of visceral adipose tissue, oxidative stress and inflammation and modulation of the gut microbiome^(23)^. These pathophysiological processes have also been named in the context of depression and obesity^(10,11)^. Also, a positive influence of weight loss programmes on depressive symptoms and mental health in people with overweight and obesity is described^(24,25)^.

The concept that it is not only *what* is eaten that counts but also *how* it is eaten is embodied as mindful eating. Mindful eating is defined as persistent attention to sensory elements of eating, a non-judgemental awareness of thoughts and feelings while eating, awareness of hunger and satiety, finding a quiet place and avoiding distractions^(26,27)^.

A mediator analysis shows a direct effect of mindful eating on depression severity. The effect of three of four mindful eating domains (focused eating, eating with awareness, eating without distraction) on depression severity is mediated neither by adherence to the Mediterranean diet nor by energy intake independent of BMI^(28)^. In a systematic review, positive effects on binge eating, emotional eating and eating in response to external stimuli through increased mindfulness are described^(29)^. A meta-analysis shows that mindfulness interventions have a positive effect on eating behaviour, depression and weight loss in people with overweight and obesity^(30)^.

In summary, it can be said that depression and obesity are highly prevalent diseases that negatively impact each other and are correlated in various social and pathophysiological ways. People affected are in need of long-term, effective treatment, but therapy options are scarce and sometimes hardly accessible. A positive effect of the Mediterranean diet, as well as mindful eating, on both conditions has already been shown. However, their synergetic effects remain unclear. The planned study now investigates their separate and combined effect in the target group of people with co-morbid depression and obesity. It provides important insights not only on the effectiveness of the different interventions but also into potential synergetic effects and therefore contributes to improving available therapy options for people suffering from depression and obesity.

## Objective

The primary aim of this study is to investigate the efficacy of the Mediterranean diet and/or mindful eating on depression severity in people with clinically diagnosed major depressive disorder and obesity. The study design allows separate and combined investigation of physiological and psychological aspects of a nutritional intervention in order to identify possible synergistic effects. In secondary analyses, moderator and mediator analysis, microbiome analysis and economic evaluation of the intervention will be conducted. The feasibility of the intervention, as well as the requirements and needs of the target group in relation to the interventions, will be examined in a qualitative approach.

## Methods

### Study design

The study is designed as a 2 × 2 factorial randomised controlled intervention trial. The four intervention groups consist of Mediterranean diet (yes/no) and mindful eating (yes/no). The intervention lasts 12 weeks, followed by a 12-week follow-up. Outcome assessment and data analysis are conducted by blinded study personnel.

### Ethical approval, trial registration and trial status

The study follows Good Clinical Practice principles in line with the Declaration of Helsinki^(31)^. Ethical approval was obtained by the ethics committee of the University of Tübingen (331/2024BO1). The trial was prospectively registered on Clinical Trials (registration number NCT06621394). Registration follows the suggestions of the WHO Trial Registration Data Set. The study is starting recruitment in October 2024 (first patient in) and is expected to end in December 2026 (last patient out).

### Study setting

The study will take place at the Bosch Health Campus in Stuttgart, Germany. The interventions will be conducted in five personal one-on-one appointments.

## Recruitment and study enrolment

**Fig. 1.**
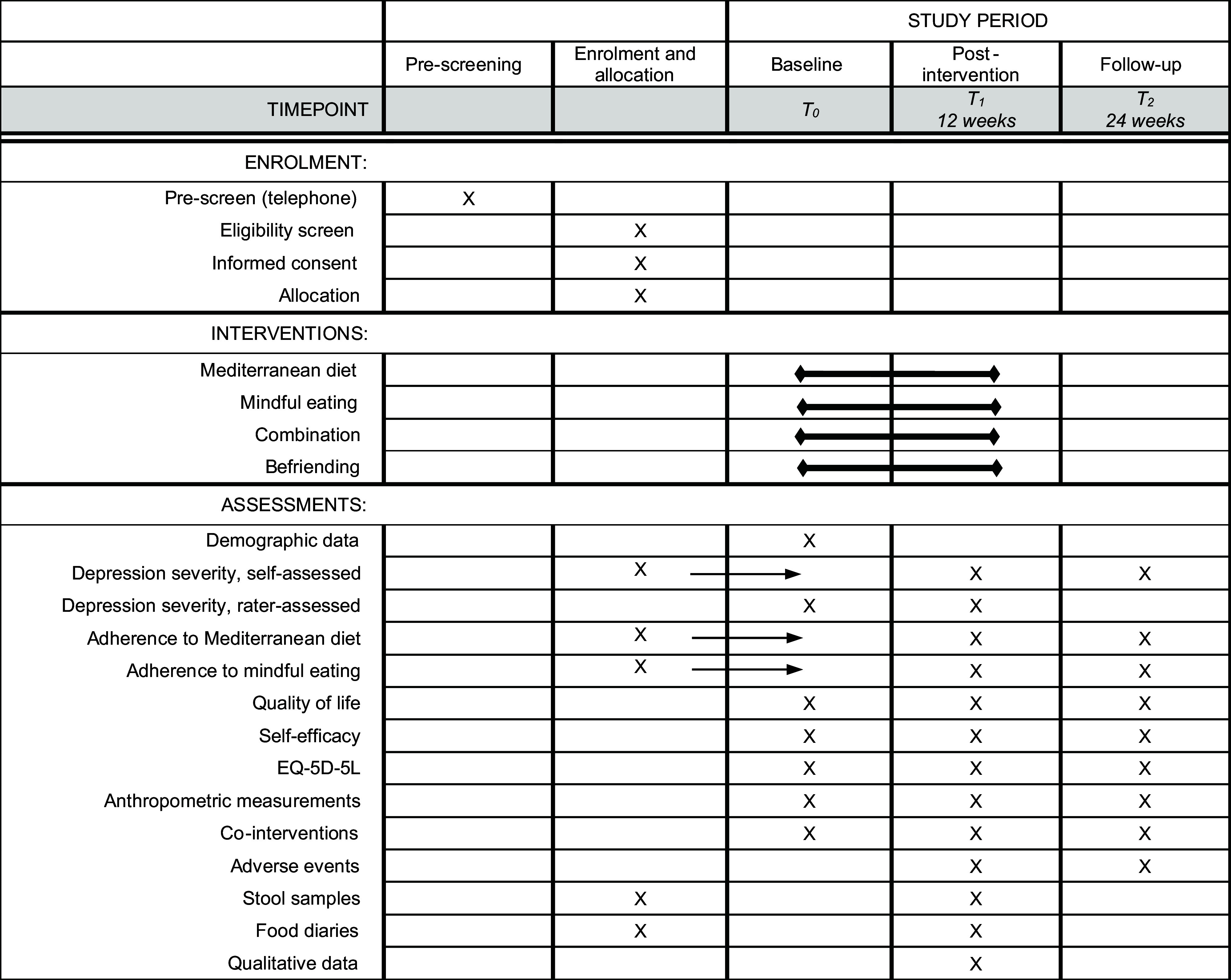
Overview of enrolment, interventions and assessments. EQ-5D-5L, European Quality of Life 5 Dimensions 5 Level.

Participants will be recruited in the Department of Psychosomatic Medicine of the Robert Bosch Hospital, Stuttgart, and via social media. Information about the study can be obtained from a website. People interested in participation can contact the study centre. After checking for the main inclusion criteria by phone or digitally (pre-screening), they will be invited for the inclusion examination and will provide written informed consent. In case of inclusion, an appointment for baseline measurement and the first intervention appointment will be made. An overview of enrolment, intervention and assessment is given in Fig. 1.

For microbiome analysis, additional samples of healthy controls (i.e. free of depression and obesity) will be collected. Inclusion of participants without depression and obesity allows better interpretation of the results from microbiome analysis and its potential changes over the intervention. They will be recruited from employees of the Robert Bosch Hospital via email. Healthy controls will come to the study centre to obtain written informed consent and fill in questionnaires. In case of inclusion, they will be handed the necessary materials for collecting the stool sample. When the sample is handed back in, participants will be reimbursed with a 20€ voucher. They will not receive any intervention.

## Study population

Study participants are adults with major depressive disorder and obesity.

### Inclusion criteria

- Age ≥ 18 years
- Diagnosis of major depression
- At least moderate depression severity: Beck Depression Inventory – II (BDI-II) ≥ 20
- Obesity: BMI ≥ 30 kg/m^2^
- Stable co-intervention: no change in the type, dosage or frequency of antidepressant medication and/or psychotherapy 4 weeks before the study and no plans to change in the next 12 weeks
- Medium to low adherence to the Mediterranean diet: Mediterranean Diet Adherence Score < 10
- Medium to low adherence to mindful eating: Mindfulness Eating Inventory < 5·13

*Given no previous cut-off scores, for the purpose of this study, we have defined the cut-off value of < 5·13 as the inclusion criterion corresponding to the mean value of the norm population plus 2 sd* ^(27)^.

### Exclusion criteria

- Diseases of the gastrointestinal tract that do not allow adequate implementation of the intervention (e.g. irritable bowel syndrome, colorectal carcinoma)
- Metabolic diseases with a strong impact on intervention (e.g. type 1 diabetes mellitus, chronic kidney disease)
- Severe food allergies and intolerances that do not allow adequate implementation of the intervention
- Diagnosed, current psychological co-morbidities (bipolar disorder, eating disorder, personality disorder, psychosis)
- Intake of antibiotics during the last 3 months
- Diagnosed, current substance abuse
- Pregnancy or breastfeeding
- Acute suicidal ideation
- Unable to participate or to complete questionnaires

### Healthy controls for microbiome analysis

For healthy controls, the following inclusion and exclusion criteria are defined:

#### Inclusion criteria

- Age ≥ 18
- BMI 20–30 kg/m^2^

#### Exclusion criteria

- Depression (BDI-II ≥ 20) or other psychological co-morbidities (bipolar disorder, eating disorder, personality disorder, psychosis)
- High adherence to Mediterranean diet (Mediterranean Diet Adherence Score ≥ 10)
- High adherence to mindful eating (Mindfulness Eating Inventory ≥ 5·13)
- Severe chronic metabolic disease or disease of the gastrointestinal tract (e.g. irritable bowel syndrome, diabetes mellitus type 1)
- Intake of antibiotics during the last three months
- Substance abuse
- Pregnancy or breastfeeding

## Randomisation

After inclusion, participants are randomised to one of the four intervention groups. Randomisation is performed using a randomisation list with varying block lengths (4, 8, 12) created by the programme R. Group allocation is carried out by members of the research staff via RedCap. Therefore, allocation concealment is given. Severity of obesity (obesity grade 1: BMI 30–35 kg/m^2^; obesity grade 2–4: BMI ≥ 35 kg/m^2^) and severity of depression (moderate depression: BDI-II 20–28; severe depression: BDI-II ≥ 29) are defined as strata. Participants are assigned to one of the four intervention groups in a 1:1:1:1 ratio. Participants will be informed about the group assignment at their first intervention appointment.

## Sample size calculation

Sample size calculation was carried out with the programme R Version 4.4.0, package ‘pwrss’ version 0.3.1. It is based on effect sizes of comparable interventions of the Mediterranean diet^(32)^ and mindful eating^(33)^ on the primary outcome of the study (BDI-II score). Using a conservative approach, we have used the lower effect size of d = 0·86 of the mindfulness intervention (Mediterranean diet: d = 1·7) to compute the required sample size. Considering a statistical power of *β* = 0·8 and *α* = 0·05, as well as a 20 % dropout rate for ANCOVA with main and interaction effects and baseline scores as covariate, *n* 64 subjects need to be recruited, that is, sixteen participants in each group.

For microbiome analysis, samples of *n* 32 healthy controls will additionally be analysed, representing the same number of participants receiving each intervention.

## Intervention

**Fig. 2.**
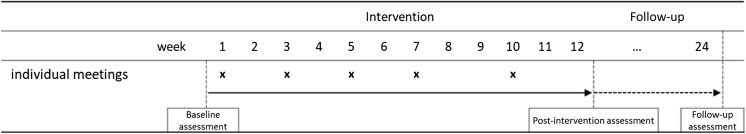
Study timeline.

The intervention takes place over a 12-week period followed by a 12-week follow-up. Each intervention consists of five individual appointments (week 1, 3, 5, 7, 10; duration: first appointment 60 min, following 45 min). The study timeline is presented in Fig. 2.

### Group 1: Mediterranean diet

Participants in this group are asked to change their food intake towards the Mediterranean diet. The Mediterranean diet is composed of at least two portions of vegetables and fruit, (whole-grain) cereal products and olive oil as the main source of fat in every meal. Two portions of dairy products and olives, nuts or seeds are consumed daily and at least two portions of legumes and fish, two to four eggs and two portions of white meat each week. The intake of sweets and red and processed meat is limited to twice and once a week, respectively^(34)^. Aspects such as sociability, seasonality and physical activity, which are part of some definitions of the Mediterranean diet^(35)^, are not addressed. The intervention is based on a food diary, which the participant will be filling out for 3 days in advance. Like this, personnel dietary preferences and habits (i.e. vegetarian diet, aversion to certain foods) can be considered throughout the intervention to increase practicability and long-term adherence. Personal goal setting and individual obstacles are addressed. Participants also receive information material, tips for grocery shopping and goal setting, as well as recipes and a checklist. Typical foods of the Mediterranean diet (e.g. olive oil, nuts, legumes, whole-grain pasta) are handed out at the end of each appointment.

### Group 2: mindful eating

The training for mindful eating does not address changing food choices but rather focuses on eating behaviour in terms of the way the food is eaten. It addresses eating behaviour in relation to mindfulness, appreciation, enjoyment and values. Each of the appointments provides instructions for practical implementation in everyday life. The food diary completed for 3 days in advance of the first appointment is discussed with regard to eating behaviour in order to identify individual approaches to establish mindful eating. Participants receive information material and tips for goal setting, as well as instructions and materials for practical exercises. Either materials for completing the tasks (e.g. growing sprouts at home) or a voucher of the same value as the food in groups 1 and 3 are handed out. The intervention is based on the programmes ‘Michael Pollan Teaches Intentional Eating’^(36)^ , ‘MB-EAT’ (Kristeller *et al.*, 2011^(37)^) and ‘Jon Kabat-Zinn Teaches Mindfulness and Meditation’^(38)^.

### Group 3: combination of Mediterranean diet and mindful eating

The third intervention group combines the interventions of groups 1 and 2 by providing nutritional intervention regarding the Mediterranean diet as in group 1 and addressing the same content on mindful eating as in group 2. The content and practical exercises refer to Mediterranean food. The food diary is discussed with regard to food choices, as well as the eating environment and behaviour. Participants receive the same information materials as groups 1 and 2 and the same tools for home practice as in group 2 and Mediterranean foods as in group 1 in the other sessions.

### Group 4: befriending

The control group consists of a befriending intervention, which represents a standard form of control group developed for psychological trials^(39)^, which has prior been successfully used as an active control in nutritional interventions investigating psychological outcomes^(17,19)^. Following the same scheme, it provides the same time and attention as the other intervention groups (i.e. five individual appointments), allowing for control of unspecific effects of the intervention. Positively perceived topics of everyday life and personal interests are discussed (e.g. hobbies, vacations, sports). In addition, activities such as board games and walks can be undertaken. Usage of these activities will be recorded. Like group 2, the participants also receive a voucher card with the same value as the food handed out in groups 1 and 3.

On request, all participants receive the documents of the other intervention groups after follow-up completion. No further post-trial care is foreseen. In case of reporting of adverse events, intervention will be adjusted to meet the personal needs of participants. The participants’ health has the highest priority during the whole study. In case of any serious health risks, participants may withdraw and be referred to further care.

## Outcome measures

Outcomes will be measured at baseline (T0), post-intervention (T1, 12 weeks) and after the follow-up period (T2, 24 weeks) (see Fig. 1). Outcome measurement will be conducted by blinded study personnel.

### Primary outcome

As a primary outcome, depression severity is assessed using the BDI-II. This self-report questionnaire records the occurrence of various depressive symptoms in twenty-one questions on a 4-point scale. The BDI-II score ranges from 0 to 63, with higher scores indicating higher depression severity^(40)^.

### Secondary outcomes

#### Remission

Remission rates will be assessed as the number of patients who no longer show depressive symptoms, defined as BDI-II < 9.

#### Depression severity, rater-assessed

In addition to self-rating, the severity of depression is also measured by blinded outcome assessors. Therefore, the Montgomery Asberg Depression Rating Scale (MADRS) is conducted at T0 and T1. Assessment is done following the questionnaire guidelines in a face-to-face setting or via telephone by a psychologist. It consists of ten questions that are answered on a scale from 0 to 6, resulting in a total score of 0–60, with higher scores representing higher depression severity^(41,42)^.

#### Quality of life

Health-related quality of life is measured using the Short Form-36 Health Survey. It consists of thirty-six items, resulting in a mental and physical sum score, which can be further divided into the subscales vitality, physical functioning, physical pain, general health perception, physical role function, emotional role function, social role function and psychological well-being. Higher scores indicate a higher quality of life^(43)^.

#### Self-efficacy

Self-efficacy is measured using the general self-efficacy expectation scale. It measures the optimistic belief in competence in ten items and results in a total value between 10 and 40, with a higher value indicating a greater expectation of self-efficacy^(44)^.

#### BMI

Body weight is measured using the OMRON BF511 scale. Height is measured in the horizontal plane using a portable stadiometer. All measurements are conducted without shoes and in light clothing. The BMI is calculated using the formula BMI = body weight [kg]/(height [m])^2^.

#### Waist:hip ratio

The waist circumference is measured in the horizontal plane in the middle between the costal arch and the upper edge of the iliac crest, and the hip circumference is measured at the point of the largest circumference of the hip or buttock region. Both measurements are taken twice, and their mean value is calculated. If the measurements differ by more than 1 cm, they are repeated. The waist:hip ratio is calculated from the mean values.

#### Body composition

Participants’ body composition is determined using an OMRON BF511 bioelectrical impedance analysis scale. It gives information on body fat and muscle as a percentage of body weight.

### Adherence

#### Adherence to Mediterranean diet

Adherence to the Mediterranean diet is measured using the Mediterranean Diet Adherence Score (MEDAS). It consists of fourteen questions regarding the eating habits of various foods of the Mediterranean diet. The score ranges from 0 to 14, with higher values indicating higher adherence to the Mediterranean diet^(45)^.

#### Adherence to mindful eating

Mindful eating is measured using the Mindfulness Eating Inventory (MEI). The questionnaire comprises the subscales awareness of sensory experience during eating, eating in relation to awareness of stomach fullness, awareness of eating motives and triggers, connectedness, non-reactive attitude and attention focused on eating. The thirty items are evaluated on a 6-point Likert scale. The sum score is divided by the number of items, resulting in a score from 1 to 6, with higher scores indicating higher adherence to mindfulness eating^(27)^.

### Safety

To analyse the safety of the interventions, adverse events will be assessed in structured questionnaires at T1 and T2. Time point, duration (in hours or days) and strength (on a scale from 1 to 10) are recorded. In addition, participants are asked whether they see a connection between the occurrence of the adverse event and the intervention. Occurrence of serious adverse events is reported to the responsible centre for clinical studies within 24 h.

### Further data collection

Further data collected contains demographics (T0), a 3-day food diary (T0, T1) and attendance to intervention. Also, information about the usage of co-interventions and the need for psychotherapy is collected at every time point (T0, T1, T2).

### Secondary analyses

#### Mediator and moderator analysis

Weight reduction, quality of life and self-efficacy were identified as potential mediators^(24,46–48)^. Treatment adherence, assessed as the number of intervention appointments attended, is examined as a potential moderator. Additionally, exploratory analyses of mediators and moderators with group allocation as the independent variable will be conducted.

#### Microbiome analysis

To carry out a microbiome analysis, stool samples are collected at baseline and post-intervention. Metagenomic shotgun sequencing enables a taxonomic and functional analysis of the microbiome. Furthermore, the neurotransmitters tyramine, tryptamine, phenylethylamine, dopamine and serotonin are analysed. As a control, stool samples from thirty-two people without obesity and depression will be analysed.

#### Economic evaluation

Food diaries are used to calculate the costs of nutrition before and after the intervention. Using the European Quality of Life 5 Dimensions 5 Level^(49)^ questionnaire, quality-adjusted life years are calculated. These are used to carry out a cost–utility analysis from the participants’ perspective.

#### Qualitative evaluation

In addition to quantitative data, a qualitative evaluation will be conducted. At post-intervention (T1), all participants will be invited to take part in an interview. Interviews will be semi-structured and conducted by study personnel until the results have reached saturation. Interviews will cover two subject areas: (1) the perception of the intervention in terms of feasibility, difficulties in implementation, perception of study setting, planning of long-term implementation, etc., and (2) perception of effects of interventions, qualitative evaluation of changes in outcomes, that is, mood, psychological and physical well-being, self-efficacy, etc. Data analysis will be conducted using MaxQDA Software. The evaluation is based on qualitative content analysis of Mayring^(50)^, in which a defined question is analysed using a summarising analysis technique, the creation of a category system and appropriate coding in order to identify patterns, themes and connections. The analysis will be conducted by two researchers (one senior researcher with extensive experience in qualitative research and one PhD candidate) independently, with categories being discussed until consent is reached.

## Statistical analysis

The statistical analysis will be carried out using the programme R version 4.4.0. As a significance level, p ≤ 0·05 is defined. Data will be analysed as an intention-to-treat analysis. To handle missing data, the ‘missForest’ package in R will be used. This package implements a non-parametric imputation method based on random forests. This approach iteratively imputes missing values using a predictive model that leverages relationships among observed data while accounting for both continuous and categorical variables. Default settings for the algorithm will be used, which automatically selects the optimal number of trees and variables per split to maximise imputation accuracy. The primary outcome parameter is analysed confirmatory using a two-factorial ANCOVA, in which the main outcome parameter is modelled as a function of the two factors – mindful eating (yes/no) and Mediterranean diet (yes/no) – and the respective baseline values (linear covariates). Both main effects of the interventions and interactions are considered. Secondary outcomes will be exploratory (without correction for multiple testing) and analysed using the same models as the primary outcome. To estimate the clinical significance of the results, change scores and effect sizes (Hedges’ g) are calculated for between-group comparisons. The clinical relevance of the results is examined by comparing the number of participants who achieve a clinically relevant improvement of at least 5 points^(51)^ on the BDI-II between the groups at week 12 and week 24 using a logistic regression adjusted for the BDI-II baseline value (random effect). The same model is used to compare the number of patients between the groups who reach remission of depression at week 12 and week 24 (BDI-II < 9).

Mediator and moderator analyses are conducted with the PROCESS Package^(52)^. For the mediator analysis, the indirect effect of an independent variable on a dependent variable via a mediator and its significance are tested using the bootstrap method. Mediation paths can be identified and quantified with consideration of control variables. Concerning moderator analysis, the relationship between the moderator and the independent variable is analysed using product terms in the regression model. Strength and direction are determined using coefficients and statistical significance using the bootstrap method.

For economic evaluation, a cost–cost analysis is carried out to compare food costs in different study arms. Costs will be calculated according to participants’ food diaries. Further, a cost–utility analysis from the participant’s perspective is carried out. Utility is measured in quality-adjusted life years, which are calculated using the European Quality of Life 5 Dimensions 5 Level questionnaire^(49)^. Based on the costs and quality-adjusted life years, the incremental cost ratio is calculated.

## Data management

Due to the nature of nutritional intervention, participants and providers cannot be blinded. Participants will learn about their group allocation at their first intervention appointment. Outcome measurements and data analysis will be conducted blinded. Questionnaires will be filled out digitally or on paper.

Study data are collected and managed using REDCap (Research Electronic Data Capture) electronic data capture tools hosted at meDIC/University Hospital Tuebingen^(53,54)^. REDCap is a secure, web-based software platform designed to support data capture for research studies. Data management will be in accordance with data safety guidelines, and data collection and storage will be performed in accordance with Good Clinical Practice Guidelines. There will be no data monitoring committee for the study because of the unblinded and monocentric design and because interventions are delivered only in the short term, and no harm is suspected.

## Dissemination policy

The study will be published in a peer-reviewed scientific journal. Results will also be shared with participants on request. Any changes in the protocol will be communicated to the ethics committee in the form of an amendment. Results will further be presented at conferences, and efforts will be made to make results accessible to a wide audience, not only in science but also in the general population.

## Discussion

The study has the potential to add valuable new data to the uprising field of the interplay of nutrition and mental health. The role of the Mediterranean diet in depression has already been studied^(17–19,55–57)^. These studies are conducted in Mediterranean and non-Mediterranean countries and address different target groups, as those with or without a diagnosis of depression, patients at high risk for CVD or young men. By including patients with co-morbid obesity, a new and vulnerable target group is studied^(6)^. Another novelty of the study is its factorial design, which allows separate evaluation of the effect of *what* food is eaten, in the manner of the Mediterranean diet, and *how* food is eaten, displayed as mindful eating.

Various secondary outcomes such as quality of life, self-efficacy and anthropometric measures will be assessed. They were chosen based on their potential role in the effect of the interventions. The choice of a valid control group in nutritional intervention trials is challenging^(58)^. The befriending control group was already successfully established in similar trials^(17,19)^ and allows for control of unspecific factors such as time, therapist relationship and expectations, which may play an important role in nutrition interventions and when studying mental health outcomes.

Changing eating behaviour is very complex. Many efforts are made to ease adherence. Food hampers, as well as many different supporting materials are provided. Further, the one-on-one setting allows focusing on the individual preferences and needs of participants.

If participants are interested, they will receive all printed materials of the other study groups on request after finishing participation. Adherence, dropouts and adverse events will be systematically analysed. In combination with the qualitative evaluation of the study, important findings regarding feasibility and implementation in a real-life setting will be gained.

The study evaluates the efficacy of the Mediterranean diet and mindful eating on depression severity and contributes to a better understanding of their mechanisms of action. By including participants with co-morbid depression and obesity, it serves a new and very important target group. The results may be seen as an important contribution to improving the supply situation for people suffering from obesity and depression.

## Supporting information

Moosburner et al. supplementary material 1 Moosburner et al. supplementary material

Moosburner et al. supplementary material 2 Moosburner et al. supplementary material

## Supplementary material

The supplementary material for this article can be found at https://doi.org/10.1017/S0007114525105849
